# Cerebrospinal Fluid Neurotransmitters, Pterins, Folates and Amino Acids in Paediatric Onset Epilepsies: A Tertiary Centre Retrospective Cohort Study

**DOI:** 10.3390/children12111514

**Published:** 2025-11-09

**Authors:** Mario Mastrangelo, Claudia Carducci, Filippo Manti, Giacomina Ricciardi, Rossella Bove, Francesco Pisani, Vincenzo Leuzzi

**Affiliations:** 1 Department of Woman/Child Health and Urological Sciences, Sapienza University of Rome, 00185 Rome, Italy; 2 Unit of Child Neurology and Psychiatry, Department of Neuroscience/Mental Health, Azienda Ospedaliero Universitaria Policlinico Umberto I, 00161 Rome, Italy; filippo.manti@uniroma1.it (F.M.); francesco.pisani@uniroma1.it (F.P.); 3 Department of Experimental Medicine, Sapienza-University of Rome, 00185 Rome, Italy; claudia.carducci@uniroma1.it; 4 Department of Human Neuroscience, Sapienza-University of Rome, 00185 Rome, Italy; giacomina.ricciardi@uniroma1.it (G.R.); rossella.bove@uniroma1.it (R.B.);

**Keywords:** cerebrospinal fluid, neurotransmitter disorders, biogenic amines, amino acids, folates, pterins

## Abstract

**Objectives:** To investigate the clinical value of cerebrospinal fluid (CSF) testing for biogenic amine, pterins, amino acids, and folates in paediatric onset epilepsies. **Methods:** Retrospective clinical and biochemical phenotyping of patients with epilepsy who underwent diagnostic CSF measurement of monoamine neurotransmitters, pterins, folates, and amino acids between 2009 and 2022. **Results:** The studied cohort included 123 patients with epilepsy (mean age at the procedure: 4.54 ± 3.65 years). The diagnostic yield for primary neurotransmitter disorders was 1.68% and zero for inherited amino acid and folate metabolism disorders. Patients with higher seizure frequency showed higher levels of CSF homovanillic acid (HVA) and HVA/5-hydroxyindolacetic acid (5HIAA) ratio. Lower levels of 3-O-methyldopa (3-OMD) were found in patients with co-occurring neurodevelopmental disorders, and lower levels of biopterin, 3-methoxy-4-hydroxyphenylglycol (3-MHPG) and 5-methyltetrahydrofolate (5-MTHF) in those with movement disorders. Significantly lower CSF glutamine levels were found in patients receiving antiseizure medications as polytherapy. Patients with a history of status epilepticus had significantly lower levels of CSF aspartic acid, glycine, leucine, ornithine, and valine, and higher levels of CSF serine. **Conclusions:** CSF analysis disclosed differences in the concentrations of various metabolites that might be related to the severity of the epilepsy, the presence of comorbid conditions, and medications.

## 1. Introduction

Cerebrospinal fluid (CSF) analysis is often a critical component of the diagnostic work-up for neurogenetic and neurometabolic disorders associated with epilepsy, as the early identification of specific pathological patterns can significantly improve patient outcomes [1]. The early onset of seizures combined with other neurological signs and symptoms (e.g., neurodevelopmental impairments, movement disorders, or intellectual disabilities) may mandate a lumbar puncture during the initial diagnostic evaluation of children with epilepsy to prevent delays in initiating specific treatments [1]. These diagnostic paradigms may change soon with the development and dissemination of ultrafast exome and genome sequencing, which will likely counterbalance the current temporal advantage that some biochemical investigations still offer in clinical practice [2].

CSF abnormalities might reflect the underlying primary pathogenic mechanisms of specific diseases or serve as biomarkers of secondary metabolic alterations induced by epileptogenic processes [1].

The present study retrospectively investigated the metabolic CSF profiles (including biogenic amines, pterins, amino acids, and folates) and their pathophysiological significance in a cohort of patients with paediatric-onset epilepsies referred to our tertiary centre over a wide timeframe.

## 2. Patients and Methods

**Figure 1:**
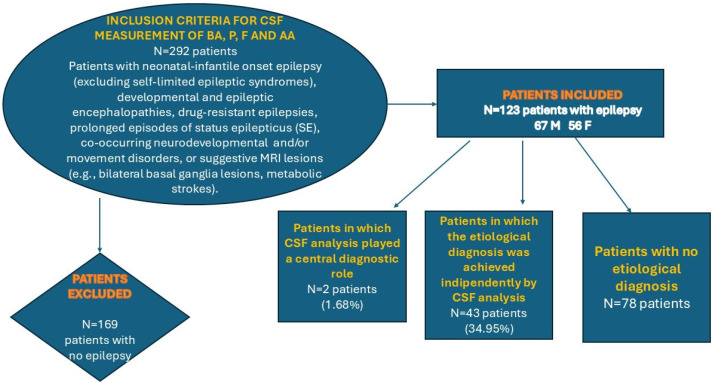
A flow diagram summarising the indications for the CSF measurement of biogenic amine, pterins, folate, and amino acids and the proportion of patients with epilepsy in which the etiological diagnosis was achieved with and without the contribution of lumbar puncture. LEGEND: BA = biogenic amine, P = Pterins, F = folates, AA = amino acids, M = males, F = females, N = number of patients.

Patients with epilepsy who were referred to our centre and underwent lumbar puncture for the measurement of CSF monoamine neurotransmitters, pterins, folate, and amino acids between 2009 and 2022 were included in this study (Figure 1).

The primary indications for performing a lumbar puncture were defined within an institutionally approved diagnostic protocol and included neonatal/infantile onset (excluding self-limited epileptic syndromes), developmental and epileptic encephalopathies, drug-resistant epilepsies, prolonged episodes of status epilepticus (SE), co-occurring neurodevelopmental or movement disorders, or suggestive MRI lesions (e.g., bilateral basal ganglia lesions, metabolic strokes) (Figure 1).

An extensive chart review and an ad hoc digitised data sheet were used to collect information on the following variables: age at seizure onset, age at lumbar puncture, seizure types and frequency, seizure-to-lumbar puncture interval, features of pharmacological antiseizure treatment, presence of drug resistance, ascertained history of SE, co-occurrence of movement or neurodevelopmental disorders, and, if available, molecular genetic diagnosis.

CSF samples were collected via lumbar puncture performed in the early morning while patients were fasting and sedated with midazolam and propofol. The samples were immediately frozen at the bedside using dry ice and stored at −70 °C [3]. Amino acids and pterins were quantified by high-performance liquid chromatography with fluorescence detection [4,5], whereas monoamine neurotransmitter metabolites and 5-methyltetrahydrofolate (5-MTHF) were measured by HPLC electrochemical detection [3].

All statistical analyses were conducted using IBM SPSS Statistics version 25.0 (SPSS Inc., Chicago, IL, USA). A probability value of *p* < 0.05 was chosen as the significance level for all tests; however, due to multiple comparisons, *p*-values were adjusted according to the Benjamini–Hochberg method, adopting an adjusted *p*-value *<* 0.05 as significant. Quantitative data are presented as mean ± standard deviation (SD). Normality was assessed using the Kolmogorov–Smirnov test. Comparisons were carried out using the independent samples *t*-test for continuous variables and the chi-square test for categorical variables. A multivariate analysis of variance (MANOVA) was realised to evaluate the effects of CSF biomarkers on the dependent variables (movement disorders, global developmental delay/intellectual disability, and status epilepticus). The multivariate models included the following covariates: age, sex, seizure-to lumbar puncture interval, number of antiseizure medications, drug resistance, molecular genetic diagnosis, and co-occurrence of neurodevelopmental or movement disorders. Univariate analysis of variance (ANOVA) was realised to identify which dependent variables contributed to the significant multivariate effects.

Written parental informed consent was obtained for all the patients included in the study. The study was approved by the Territorial Ethics Committee Latium Area 1 (approval code 7125-0292/2023; date of approval 29 March 2023).

## 3. Results

### 3.1. Cohort Composition

During the analysed timeframe, 123 patients with epilepsy (67 males; 56 females) underwent a diagnostic lumbar puncture for the measurement of monoamines, pterins, folates, and amino acids. Table 1 and Table S1 summarise the main demographic, molecular genetics, and clinical features, including the epilepsy phenotype, of the entire cohort.

A definitive etiological diagnosis was obtained in 45 patients (36.5%). These included single gene-related diseases in 36 patients, pathogenic copy number variants in 8, and anti-NMDAR encephalitis in 1 (Table 1 and Table S1). The disease-causing genes were involved in cell cycle regulation (20 patients), channelopathies (10 children), transportopathies (6), and intermediate metabolism abnormalities (5 patients) (Supplementary Table S1). *SLC2A1* and *SCN1A* were the genes with the highest number of detected pathogenic variants (respectively, *n* = 4 and *n* = 3 patients; Supplementary Table S1).

The mean age at seizure onset was 2.19 ± 0.41 years (range: 3 days–15 years; Table 1 and Table S1). The predominant seizure types were focal motor/focal motor to bilateral tonic–clonic seizures (63 patients). Specific epileptic syndromes were defined in 21 patients, with Infantile Spasms syndrome being the most common (12 patients) (Table 1 and Table S1). Seizure frequency was multi-daily in 67 patients, weekly/multi-weekly in 26, monthly/multi-monthly in 16, and sporadic in 18 (Table 1 and Table S1). Episodes of status epilepticus (SE) occurred in 16 patients (Table 1 and Table S1). Drug resistance, defined by ILAE criteria, was observed in 69 patients (Table 1 and Table S1) [6].

An associated neurodevelopmental disorder was assessed in 101 patients. This included Global Developmental Delay (n = 35), various degrees of Intellectual Disability (mild n = 7, moderate n = 3, severe n = 16, unspecified n = 27), language disorder (n = 5), learning disorders (n = 2), developmental coordination disorder (n = 4), and ADHD (n = 2). A delay in developmental milestones was reported in 86 patients, while intellectual disability was assessed in 51 patients. Movement disorders occurred in 60 patients, predominantly hyperkinetic types (n = 44).

Brain MRI showed sufficiently specific diagnostic patterns to guide the etiological diagnosis in four patients (affected by Canavan disease, Metachromatic leukodystrophy, Menkes disease, and *FOXG1* encephalopathy, respectively).

### 3.2. CSF Metabolic Profiles

CSF sampling was performed at a mean age of 4.54 ± 3.65 years. The examination included biogenic amine, pterin, and amino acid measurement in all patients, while 5-methyltetrahydrofolate (5-MTHF) was assessed in 58/123 patients.

Seizure frequency at the time of lumbar puncture and seizure-to-lumbar puncture interval were variable (Table 1 and Table S1). Seizures were observed within 24 h prior to CSF collection in 67/123 participants (54, 47%), and within a few days to one month in 42 cases (Table 1 and Table S1).

At the time of CSF sampling, 79/123 subjects were receiving antiseizure medications (monotherapy in 46 patients and polytherapy in 32; Supplementary Table S1). The most common agents were valproic acid (n = 33), phenobarbital (n = 23), and levetiracetam (n = 15) (Table 1). Two participants (Patients 110 and 113 in Supplementary Table S1) were also receiving specific antiparkinsonian treatment (L-Dopa, selegiline, and folinic acid) following a newborn screening diagnosis of DHPR deficiency. In these two patients, dopaminergic supplementation did not result in significant benefits on either epilepsy or biogenic amine levels.

A relevant reduction in HVA was detected in two patients with DHPR deficiency (Patients 110 and 113 in Supplementary Table S1), a 1-month-old female with *SCN2A*-related developmental and epileptic encephalopathy (Patient 1 in Supplementary Table S1), a 4-year-old male with neuronal ceroid-lipofuscinosis type 2 (Patient 80 in Supplementary Table S1), and four patients without an etiological diagnosis (Patients 35, 78, 81, and 84 in Supplementary Table S1).

CSF 3-MHPG levels were more than two times higher than the upper limit of the reference range for age in six patients (Patients 25, 33, 38, 48, 102, and 109 in Supplementary Table S1).

CSF 3-OMD increase was observed in 14 out of 20 children under 3 years of age, irrespective of the diagnosis. In the only patient with a large deletion involving sodium channel cluster genes, the detected value was two times the upper reference level (Patient 18). The increased levels of this metabolite were influenced by concomitant L-Dopa treatment in the two DHPR deficiency patients (Patients 110 and 113 in Supplementary Table S1).

A significant decrease in 5-HIAA and a consistent increase in 5HTP were detected in a patient with Rett syndrome (Patient 43 in Supplementary Table S1).

The highest deviation from the reference range for the HVA/5-HIAA ratio was observed in a patient with Infantile Spasms syndrome of undefined aetiology and a patient with Neuronal Ceroid Lipofuscinosis type 2 (Patients 21 and 80 in Supplementary Table S1).

The most consistent increase in neopterin was recorded in Patient 3 (non-established aetiology) and Patient 64 (*PRRT2*-related epilepsy), while the highest deviation from the reference range for biopterin was reported in Patient 9 in Supplementary Table S1.

Increased levels of CSF phenylalanine and normal levels of tyrosine were found in the two patients with DHPR deficiency (Patients 110 and 113 in Supplementary Table S1).

Cerebral folate deficiency was demonstrated in nine patients (one with *KCNH1*-related developmental and epileptic encephalopathy, one with DHPR deficiency, one with Glut1 deficiency, one with a *PRRT2* pathogenic variant, and five with no established etiological diagnosis). A non-specific increase in CSF 5-MTHF was detected in fifteen patients (two of whom had an extended deletion of sodium channels cluster genes, one a *PRRT2*-related disorder, one a Glut1 deficiency, and one a *FOXG1* encephalopathy) (Supplementary Table S1).

### 3.3. Clinical–Biochemical Correlations

The within-group comparison highlighted significantly higher levels of HVA (453 ± 171.8 versus 239 ± 102.3 nmol/L) in the age-range 6–12 years and a higher HVA/5-HIAA ratio (1.21 ± 0.92 versus 0.84 ± 0.58; Table 2) across all age ranges in patients with a higher seizure frequency. Patients receiving polytherapy more frequently showed lower 5-HIAA values in the 3–5-year age range, while 3-MHPG was higher in patients with drug-resistant epilepsies between 6 and 12 years (Table 3).

No relevant differences in metabolite levels were detected between patients with predominantly focal and predominantly generalised seizures (Table 2).

Patients with episodes of SE presented with significantly lower levels of CSF aspartic acid, glycine, leucine, ornithine, and valine, and a higher CSF serine level (Table 3).

A significantly lower CSF glutamine level (*p* < 0.01) was found in subjects receiving pharmacological antiseizure treatment (who also had lower levels of 2-aminobutyric acid and 5-MTHF) and in patients under polytherapy. In patients with drug resistance, a trend towards significance was observed even if it was not confirmed after Benjamini–Hochberg correction (Table 3).

The within-group comparison of subjects with and without a movement disorder disclosed a significant difference in 5-MTHF (33.40 ± 68.98 versus 90.29 ± 87.43; *p* = <0.01), 3-MHPG (age range 3–5 years: *p* = 0.05) and biopterin levels (5.05 ± 2.25 vs. 6.26 ± 2.76 nmol/L; *p* = 0.010) (Table 4).

The within-group comparison between subjects with and without an associated neurodevelopmental disorder disclosed a significant decrease in CSF 3-OMD (*p* = 0.009 in the age-range 3–5 years; Table 3) and an increase in aspartic acid (*p* = 0.022) and proline levels (*p* = 0.016) (Table 4).

Multivariate analyses (MANOVA) revealed a significant main effect of CSF biopterin levels on the dependent variables investigated, [Wilks’ Lambda = 0.885, F (3, 77) = 3.33, *p* = 0.024, η^2^ₚ = 0.115 (95% CI 0.02, 0.23)]. In addition, the CSF aspartic acid level had a strong multivariate effect [Wilks’ Lambda = 0.843, F (3, 77) = 4.79, *p* = 0.004, η^2^ₚ = 0.157 (95% CI 0.05, 0.28)]. Univariate ANOVAs highlighted that CSF biopterin levels had a significant effect for both the presence of a movement disorder [F (1, 79) = 7.14, *p* = 0.009, η^2^ₚ = 0.083 (95% CI 0.02, 0.16)], and status epilepticus [F (1, 79) = 4.29, *p* = 0.042, η^2^ₚ = 0.051 (95% CI 0.00, 0.10)]. A lower CSF aspartic acid level was significantly observed with the occurrence of a global developmental delay/intellectual disability [F (1, 79) = 7.12, *p* = 0.009, η^2^ₚ = 0.083 (95% CI 0.04, 0.25), and status epilepticus, F (1, 79) = 7.69, *p* = 0.007, η^2^ₚ = 0.089 (95% CI 0.04, 0.26)].

## 4. Discussion

The analysis of the herein reported cohort highlighted two main aspects regarding the measurement of CSF biogenic amines, pterins, amino acids, and folates in patients with epilepsy: a relatively low diagnostic yield and the potential role of specific biochemical parameters as biomarkers of clinical severity and outcome (Table 2, Table 3 and Table 4).

The low diagnostic yield of CSF analysis for neurotransmitter, pterin, amino acid, and folate disorders in epilepsy has previously been reported, with some variations depending on the age range [1,7,8,9,10]. This low yield is strictly correlated with the rarity of the associated neurometabolic diseases [7]. For instance, in a cohort of 323 infants under 12 months (292 of whom had epileptic seizures), no subjects were found with primary biogenic amine or folate disorders [7]. In another cohort of 205 patients with developmental and epileptic encephalopathies under the age of three years, only one patient was diagnosed with FOLR1 deficiency, while a smaller, similar-age cohort yielded no diagnoses [8,9]. The diagnostic yield reached higher values (up to 5.3%, with 4% being inherited neurotransmitter diseases) in cohorts where epilepsy coexisted with movement disorders [1,10]. A yield of 25.8% was reported in a cohort where additional mandatory indications for lumbar puncture were included (e.g., diurnal fluctuation of symptoms, autonomic signs, and neurodevelopmental disorders) [1,10]. These last presentations likely represent the best indications for measuring CSF neurotransmitters due to the correlated therapeutic implications, which may involve treatments such as dopamine mimetics, pyridoxine, and folate supplementation, or even gene therapy for disorders like Aromatic L-amino acid decarboxylase (AADC) deficiency [11,12]. Eleven published cases of patients presenting with epilepsy and secondary CSF neurotransmitter abnormalities underwent dopamine and serotonin replacement therapies; five had pathogenic variants in known disease-causing genes (*SCN2A*, *KIAA2022*, *MECP2*, *FOLR2*, and *FGF13*), and six had unknown aetiologies [6,13]. Transient improvements in seizure control were observed in seven patients, with additional beneficial effects on attention span, interaction, task focus, anxiety, and aggression [6,13]. However, these positive effects were not confirmed in the two patients with DHPR deficiency in our cohort (Patients 110 and 113 in Supplementary Table S1), who were receiving L-dopa, selegiline, and folinic acid.

Despite remaining within the normal range, a trend toward higher HVA values was found in patients aged 6 to 12 years with more frequent seizures (Table 2). A decrease in HVA has been previously reported in children with epilepsy, particularly in infants with neonatal-onset developmental and epileptic encephalopathies and Infantile Spasms syndrome [6]. The tendency for elevated HVA levels in this older subgroup of our cohort might reflect age-dependent, seizure-related recruitment of dopamine networks [12]. This phenomenon could stem from a possible over-activation of neuronal cell death cascades involving D1 receptors, glutamate networks, PKA/ERK, and mTOR signalling, coupled with a downregulation of D2 receptors [12]. The involvement of these epileptogenic mechanisms is supported by both in vitro studies using animal models of temporal lobe epilepsy and the higher consumption of the dopamine neurotransmission regulators, biopterin and 5-methyltetrahydrofolate, observed in the subgroup of our patients with concomitant movement disorders [14].

The elevated HVA/5-HIAA ratio observed in patients with higher seizure frequency was likely secondary to a concomitant increase in HVA concentrations (Table 2). An alternative mechanism contributing to the increased HVA/5-HIAA ratio might involve impaired serotonergic regulation of dopamine activity through glutamatergic co-transmission and 5HT3 receptors [15,16,17]. These recent seizure-related changes might carry greater prognostic significance because the neuronal networks involved are the same as those implicated in SUDEP [16]. Similar prognostic implications might be associated with the possible serotoninergic and noradrenergic dysregulation behind the lower values of 5-HIAA and the higher values of 3-MHPG in patients under polytherapy and drug-resistance in the subgroups of our cohort including late infancy and school-aged children (Table 3) [16].

The lower levels of cerebrospinal fluid (CSF) 3-OMD observed in the 3- to 5-year-old subgroup with co-occurring neurodevelopmental disorders warrant attention and might have several explanations. 3-OMD is a surrogate metabolite of levodopa; thus, its low concentration might suggest reduced activity of tyrosine hydroxylase (TH), the rate-limiting enzyme in dopamine synthesis [18]. We consider this TH hypothesis less probable for the overall cohort, as indicated by the predominantly normal values of 3-OMD (the surrogate CSF metabolite of dopamine) reported in the entire study population [18]. However, in the context of severe neurodevelopmental disorders, such as those present in the enrolled patients, a low 3-OMD level might point to a general impairment in the development of the prefrontal dopaminergic system [18]. This system is crucial for the emergence of executive functions and normal intellectual development in children [18]. A recent pilot study involving 390 patients with neurodevelopmental disorders did not detect abnormal 3-OMD levels in dried blood spots [19]. However, that study was primarily designed to identify an increase in the metabolite as a diagnostic marker for AADC deficiency, rather than a reduction [19]. Consequently, no data were reported regarding the full range of 3-OMD values, limiting its relevance to the current findings [19].

Significant differences in the levels of various amino acids (e.g., aspartic acid, ornithine, leucine, valine, glycine, and serine; Table 2) were detected in the small proportion of patients in this cohort presenting with status epilepticus (SE). Specifically, the significantly lower levels of aspartic acid (Table 2) are consistent with previous preclinical findings from a lithium-pilocarpine-induced SE mouse model, which reported similar changes in rat hippocampal micro dialysates [20]. In the same study, increased levels of the same amino acid were detected in rats that developed a chronic epileptic condition but were outside the acute phase [18]. These differences might implicate a differential recruitment of the aspartate-glutamate carrier across the acute and chronic stages of epilepsy, potentially resulting in increased aspartate consumption (acute stage) or increased aspartate synthesis (chronic stage), respectively [21]. The increased recruitment of metabolic cascades involving aspartate and glutamate metabolism might also account for the concurrent lower levels of CSF ornithine observed in our cohort (Table 2) [22]. The lower levels of leucine (Table 1) might suggest a shift from an anabolic to a catabolic state, possibly resulting from the substantial energy consumption associated with protracted epileptic seizures [23]. In another mouse model, the administration of L-leucine resulted in the protection against kainic-acid-induced seizures, suggesting that its low CSF levels might facilitate a lower seizure threshold [24]. The concurrent lower levels of valine and glycine observed in the same patient group (Table 1) might underlie the lack of other protective antiseizure actions (e.g., reduced activation of GABA networks and reduced inhibition of glycinergic neurotransmission on NMDAR-mediated excitotoxicity, respectively) [25,26]. The higher levels of CSF serine observed in our cohort (Table 2) might be associated with the activation of other epileptogenic mechanisms, including a) direct overstimulation of NMDA receptors, which could result in the upregulation of associated excitotoxic cascades, and b) increased recruitment of serine racemase and subsequent enhanced D-serine synthesis from L-serine [27]. These pathways were hypothesised to occur through the impaired phosphorylation of Extracellular Signal-regulated Kinase (ERK), leading to a higher neurotoxic effect [27].

Interestingly, higher levels of CSF aspartic acid in the present cohort were more frequent in patients with neurodevelopmental disorders (Table 1). An intriguing pathogenic link between aspartate and neurodevelopmental impairment might involve the process of racemization from L- to D-aspartate [21,28]. However, this hypothesis is difficult to confirm, as D-aspartate is not routinely measurable in CSF [21,28]. D-aspartate is crucial for enhancing NMDA receptor-dependent synaptic plasticity, dendritic morphology, and memory circuits in the foetal developing brain [21,28]. However, its levels are extremely low in the postnatal age [21,28]. Furthermore, its accumulation has been demonstrated in the brains of patients with Alzheimer’s disease, suggesting a potential neurotoxicity [21,28]. A similar neurotoxic effect was demonstrated in mouse models following the hippocampal injection of L-proline into rats, which resulted in the extensive destruction of pyramidal and granule cell layers [29]. Similar pathogenic mechanisms might be implicated in the higher frequency of neurodevelopmental disorders observed in patients with elevated CSF proline levels in our cohort (Table 4).

The lower levels of cerebrospinal fluid (CSF) 5-MTHF detected in children receiving pharmacological treatment were likely correlated with the known folic acid depletion induced by certain antiseizure medications, such as valproic acid, phenytoin, or carbamazepine (Table 3) [30]. The trend towards lower levels of CSF glutamine detected in patients receiving seizure medications, particularly those on polytherapy and with drug-resistant epilepsies (Table 3), do not seem to confirm previous findings that reported normal blood glutamine levels in a cohort of patients with focal symptomatic, drug-resistant epilepsy [31]. The lower glutamine levels might be the expression of altered, seizure-related activation of the astrocytic glutamate–glutamine cycle, potentially due to dysfunction of excitatory amino acid transporters (EAAT1 and EAAT2) or glutamine synthetase [31,32]. In a three-dimensional osteosarcoma model, the increased expression of several drug-resistance-related genes (Bcl2, Abcb1, Abcg2, Nanog, Sox2) was closely associated with heightened glutamine metabolism [32]. Thus, the involvement of similar mechanisms in epilepsy cannot be excluded [32].

The primary strength of this study lies in the relatively high number of patients who underwent CSF measurement of the analysed metabolic biomarkers. These patients presented with a history of epilepsy and complex comorbidities, including neurodevelopmental and movement disorders. These data have significant epidemiological value because our centre is one of only two Italian tertiary institutions capable of routinely measuring CSF biogenic amines, pterins, amino acids, and folates.

The study has also several key limitations, primarily stemming from its retrospective nature, listed as follows:

(a) Clinical heterogeneity: the retrospective design involved varying degrees of severity across the observed epilepsy phenotypes.
(b) Variable sample collection: there was heterogeneity in the patients’ ages at the time of CSF collection and the time elapsed between seizure occurrence and lumbar puncture and the precise measurement of seizure-to-lumbar puncture interval was not always feasible.
(c) Confounding factors: differences in underlying aetiologies and other factors, such as nutritional status, catabolism, and pharmacological polytherapy, may have differentially impacted the CSF levels of the measured metabolites.
(d) Different age-dependent reference ranges: this study might have underestimated the role of biogenic amines because reference ranges for most of these CSF metabolites vary across different age groups. Furthermore, the small number of patients within certain age ranges limited our ability to conduct an adequate analysis of potential correlations with clinical parameters (Table 2, Table 3 and Table 4).

These limitations might have significantly influenced the overall results of the study and need to be explored in more extended multicentre studies to support the real-world clinical utility and applicability of the above reported data.

## 5. Conclusions

Early-onset drug-resistant epilepsy and co-occurring neurodevelopmental or movement disorders should be considered an indication for lumbar puncture to measure CSF biogenic amines, pterins, folates, and amino acids.

The present study provides useful insights regarding differences in CSF metabolite levels in persons with epilepsy. These findings have potential implications for the diagnostic work-up (serving as biomarkers of clinical severity and/or outcome) and for guiding future precision medicine therapeutic strategies (specifically targeting the regulation of dopamine, serotonin, glutamate, glycine, and serine-mediated neurotransmission). Whether the observed differences in metabolite levels are the causes or the results of recurring seizures remains speculative. More systematic metabolomic studies are required to fully assess the clinical value of these metabolites beyond current applications and to determine if they can help establish a more personalised therapeutic approach for epilepsy.

## Figures and Tables

**Table 1:** Main demographic and clinical features of the reported cohort (see Supplementary Table S1 for patient-by-patient details).

**NUMBER OF PATIENTS**	123
**SEX**	67 MALES 56 FEMALES
**NUMBER OF PATIENTS WITH ETIOLOGICAL** **DIAGNOSIS**	45
**MEAN AGE AT SEIZURE ONSET**	2.19 ± 0.41 years (range: 3 days–15 years)
**NUMBER OF PATIENTS WITH SPECIFIC EPILEPTIC SYNDROMES**	Total = 21 Infantile Epileptic Spasms Syndrome = 12 Dravet syndrome = 3 Myoclonic-atonic epilepsy = 2 Lennox–Gastaut syndrome = 1 Early Infantile DEE = 1 Epilepsy of Infancy with migrating focal seizures = 1 Eyelid Myoclonia with absences = 1
**SEIZURE TYPES**	Focal impaired consciousness/Focal to bilateral tonic–clonic seizures = 63 Generalised seizures = 60
**SEIZURE FREQUENCY**	Multi-daily = 67 Weekly/multi-weekly = 26 Monthly/multi-monthly =16 Sporadic = 18
**SEIZURE TO LUMBAR PUNCTURE INTERVAL**	<24 h = 67 patients 24 h–1 month = 42 patients Not available = 14 patients
**NUMBER OF PATIENTS WITH HISTORY OF STATUS EPILEPTICUS**	16
**NUMBER OF PATIENTS WITH DRUG-RESISTANCE**	60
**NUMBER OF PATIENTS WITH CONCOMITANT NEURODEVELOPMENTAL DISORDERS**	101
**NUMBER OF PATIENTS WIYH CONCOMITANT MOVEMENT DISORDERS**	60
**ANTISEIZURE MEDICATIONS**	Valproic acid= 33 patients Phenobarbital = 23 patients Levetiracetam = 15 patients B6 = 12 patients Carbamazepine = 11 patients Clobazam = 10 patients Clonazepam = 10 patients Lamotrigine = 9 patients Topiramate = 9 patients Nitrazepam= 6 patients Vigabatrin = 5 patients ACTH = 3 patients Phenytoin = 2 patients Zonisamide = 1 patient Rufinamide = 1 patient Oxcarbazepine = 1 patient

**Table 2:** Relationships between CSF metabolic biomarkers and clinical epilepsy phenotype. The analysed metabolites, that are indicated in the first column with the different age-related reference-ranges, were compared within groups of patients defined by seizure types (focal versus generalised), seizure frequency (low versus high frequency) and occurrence of status epilepticus (absent versus present). The size of the groups was stratified according to the different age ranges. Legend: N = number of patients; m = months; y = years; NA = not applicable; FDR = Benjamini–Hochberg False Discovery Rate; * *p* < 0.05; ** *p* < 0.01.

Variables	SEIZURE TYPES (Focal vs. Generalised)			FREQUENCY OF SEIZURES (Low vs. High Frequency)			STATUS EPILEPTICUS (Absent vs. Present)			
Clinical Group	Focal Seizures	Generalised Seizures	X^2^/*p*	Low Frequency	High Frequency	X^2^/*p*	Absent	Present	X^2^/*p*	FDR-Adj *p* Value
**Number of patients**	N = 58	N = 49	-	N = 24	N = 73	-	N = 103	N = 16	-	-
**Sex**	31 M + 27 F	25 M + 24 F	0.802	10 M+ 14 F	29 M + 44 F	0.866	58 M+ 45 F	6 M + 10 F	0.186	-
**Age (months)**	174.81 ± 69.25	152.65 ± 59.34	0.081	154.46 ± 72.46	159.09 ± 62.76	0.780	163.63 ± 71.88	153.92 ± 48.99	0.533	-
**3-Methoxy-4-Hydroxyphenylglycol** **(Mean values)** **Reference range (nmol/L)** 0–3 m = 98–168 3–6 m = 51–112 6–24 m = 28–60 3–5 y = 39–73 6–12 y = 39–73 13–15 y = 28–60 >15 y = 28–60	N = 54 N (0–3 m) =2 N (3–6 m) =5 N (6–24 m) = 19 (88.7 ± 54.8) N (3–5 y) = 10 (62.3 ± 14.9) N (6–12 y) = 11 (72.5 ± 56.4) N (13–15 y) = 2 N (>15 y) = 5	N = 46 N (0–3 m) = 2 N (3–6 m) = 3 N (6–24 m) = 22 (67.5 ± 28.7) N (3–5 y) = 9 (65 ± 26) N (6–12 y) = 9 (80.6 ± 43.6) N (13–15 y) = 0 N (>15 y) = 1	NA NA 0.141 0.787 0.720 NA NA	N = 21 N (0–3 m) = 1 N (3–6 m) = 1 N (6–24 m) = 9 (78.1 ± 36.2) N (3–5 y) = 7 (59 ± 19.8) N (6–12 y) = 3 (63.1 ± 37.7) N (13–15 y) = 0 N (>15 y) = 0	N = 70 N (0–3 m) = 3 N (3–6 m) = 8 N (6–24 m) = 32 (73.5 ± 43.9) N (3–5 y) = 11 (70.6 ± 24.1) N (6–12 y) = 13 (72.7 ± 40) N (13–15 y) = 2 N (>15 y) = 1	NA NA 0.755 0.315 0.709 NA NA	N = 95 N (0–3 m) = 2 N (3–6 m) = 7 N (6–24 m) = 41 (75.6 ± 44.9) N (3–5 y) = 15 (63.7 ± 22.6) N (6–12 y) = 23 N (13–15 y) = 2 N (>15 y) = 5	N = 16 N (0–3 m) = 2 N (3–6 m) = 4 N (6–24 m) = 4 (70.3 ± 7.5) N (3–5 y) = 5 (63.3 ± 20.6) N (6–12 y) = 1 N (13–15 y) = 0 N (>15y) = 0	NA NA 0.510 0.973 NA NA NA	-
**3-O-Methyldopa** **(mean values)** **Reference range (nmol/L)** 0–3 m = 0–300 3–6 m = 0–100 6–24 m = 0–50 3–5 y = 0–50 6–12 y = 0–50 13–15 y = 0–50 >15 y = 0–50	N = 58 N (0–3 m) = 2 N (3–6 m) = 5 N (6–24 m) = 21 (35 ± 29) N (3–5 y) = 10 (20 ± 8.7) N (6–12 y) = 13 (83.9 ± 166.1) N (13–15 y) = 2 N (>15 y) = 5	N = 48 N (0–3 m) = 2 N (3–6 m) = 3 N (6–24 m) = 22 (40 ± 21.8) N (3–5 y) = 9 (31.3 ± 23.8) N (6–12 y) = 10 (29 ± 22.6) N (13–15 y) = 0 N (>15 y) = 2	NA NA 0.523 0.182 0.313 NA NA	N = 23 N (0–3 m) = 1 N (3–6 m) = 1 N (6–24 m) = 9 (46.9 ± 20.4) N (3–5 y) = 7 (25 ± 5.1) N (6–12 y) = 4 (214.9 ± 321.9) N (13–15 y) = 0 N (>15 y) = 1	N = 72 N (0–3 m) = 3 N (3–6 m) = 8 N (6–24 m) = 32 (35.5 ± 19.4) N (3–5 y) = 11 (25.6 ± 23.8) N (6–12 y) = 15 (41.6 ± 65.9) N (13–15 y) = 2 N (>15 y) = 1	NA NA 0.162 0.953 0.050 * NA NA	N = 101 N (0–3 m) = 2 N (3–6 m) = 7 N (6–24 m) = 43 (35.5 ± 18.4) N (3–5 y) = 17 (26.4 ± 18.4) N (6–12 y) = 24 N (13–15 y) = 2 N (>15 y) = 6	N = 15 N (0–3 m) = 2 N (3–6 m) = 3 N (6–24 m) = 4 (46 ± 30.5) N (3–5 y) = 5 (19.9 ± 8.7) N (6–12 y) = 1 N (13–15 y) = 0 N (>15 y) = 0	NA NA 0.544 0.459 NA NA NA	-
**5-Hydroxytryptophan** **(mean values)** **Reference range (nmol/L)** 0–3 m = 0–10 3–6 m = 0–10 6–24 m = 0–10 3–5 y = 0–10 6–12 y = 0–10 13–15 y = 0–10 >15 y = 0–10	N = 55 N (0–3 m) = 2 N (3–6 m) = 5 N (6–24 m) = 20 (7.1 ± 3.6) N (3–5 y) = 9 (5.7 ± 3.8) N (6–12 y) = 12 (23.37 ± 35.5) N (13–15 y) = 2 N (>15 y) = 5	N = 45 N (0–3 m) = 2 N (3–6 m) = 3 N (6–24 m) = 21 (6.9 ± 2.6) N (3–5 y) = 8 (6.5 ± 2.2) N (6–12 y) = 9 (9.1 ± 3.9) N (13–15 y) = 0 N (>15 y) = 2	NA NA 0.891 0.648 0.245 NA NA	N = 21 N (0–3 m) = 1 N (3–6 m) = 1 N (6–24 m) = 8 (7 ± 2.5) N (3–5 y) = 6 (6.35 ± 4.1) N (6–12 y) = 4 (30 ± 35.9) N (13–15 y) = 0 N (>15 y) = 1	N = 68 N (0–3 m) = 3 N (3–6 m) = 8 N (6–24 m) =31 (6.4 ± 2.9) N (3–5 y) = 10 (5.5 ± 1.9) N (6–12 y) = 13 (16.6 ± 31.2) N (13–15 y) = 2 N (>15 y) = 1	NA NA 0.575 0.586 0.522 NA NA	N = 96 N (0–3 m) = 2 N (3–6 m) = 7 N (6–24 m) = 42 (6.9 ± 3.1) N (3–5 y) = 15 (5.8 ± 3.1) N (6–12 y) = 22 N (13–15 y) = 2 N (>15 y) = 6	N = 15 N (0–3 m) = 2 N (3–6 m) = 3 N (6–24 m) = 4 (6.5 ± 1.8) N (3–5 y) = 5 (6.6 ± 3.1) N (6–12 y) = 1 N (13–15 y) = 0 N (>15 y) = 0	NA NA 0.742 0.644 NA NA NA	-
**5-hydroxyindoleacetic acid (5-HIAA)** **(mean values)** **Reference range (nmol/L)** 0–3 m = 189–1380 3–6 m = 152–462 6–24 m = 97–367 3–5 y = 89–341 6–12 y = 68–220 13–15 y = 68–115 >15 y = 45–135	N = 58 N (0–3 m) = 2 N (3–6 m) = 5 N (6–24 m) = 21 (225.4 ± 113.2) N (3–5 y) = 10 (127 ± 55.2) N (6–12 y) = 13 (138.1 ± 62.1) N (13–15 y) = 2 N (>15 y) = 5	N = 48 N (0–3 m) = 2 N (3–6 m) = 3 N (6–24 m) = 22 (228.7 ± 121.8) N (3–5 y) = 9 (149 ± 29.3) N (6–12 y) = 10 (173.4 ± 71.1) N (13–15 y) = 0 N (>15 y) = 2	NA NA 0.927 0.299 0.217 NA NA	N = 23 N (0–3 m) = 1 N (3–6 m) = 1 N (6–24 m) = 9 (222 ± 58.5) N (3–5 y) = 7 (148.6 ± 53.6) N (6–12 y) = 4 (103.9 ± 37.3) N (13–15 y) = 0 N (>15 y) = 1	N = 72 N (0–3 m) = 3 N (3–6 m) = 8 N (6–24 m) = 32 (222.8 ± 106.1) N (3–5 y) = 11 (136.7 ± 48) N (6–12 y) = 15 (165.7 ± 69.7) N (13–15 y) = 2 N (>15 y) = 1	NA NA 0.975 0.633 0.161 NA NA	N = 101 N (0–3 m) = 2 N (3–6 m) = 7 N (6–24 m) =43 (217.5 ± 99.1) N (3–5 y) = 17 (139.6 ± 48.3) N (6–12 y) = 24 N (13–15 y) = 2 N (>15 y) = 6	N = 15 N (0–3 m) = 2 N (3–6 m) = 3 N (6–24 m) = 4 (201 ± 82.7) N (3–5 y) = 5 (150.1 ± 51.1) N (6–12 y) = 1 N (13–15 y) = 0 N (>15 y) = 0	NA NA 0.728 0.653 NA NA NA	-
**Homovanillic** **acid (HVA)** **(mean values)** **Reference range (nmol/L)** 0–3 m = 324–1379 3–6 m = 302–845 6–24 m = 236–867 3–5 y = 231–840 6–12 y = 137–582 13–15 y = 148–434 >15 y = 98–450	N = 58 N (0–3 m) = 2 N (3–6 m) = 5 N (6–24 m) = 21 (524.4 ± 289.4) N (3–5 y) = 10 (345.7 ± 127.6) N (6–12 y) = 13 (396.5 ± 183) N (13–15 y) = 2 N (>15 y) = 5	N = 48 N (0–3 m) = 2 N (3–6 m) = 3 N (6–24 m) = 22 (604.3 ± 289.4) N (3–5 y) = 9 (414.7 ± 142.4) N (6–12 y) = 10 (416.8 ± 146.8) N (13–15 y) = 0 N (>15 y) = 2	NA NA 0.272 0.281 0.777 NA NA	N = 23 N (0–3 m) = 1 N (3–6 m) = 1 N (6–24 m) = 9 (561.5 ± 130.2) N (3–5 y) = 7 (359.1 ± 142.3) N (6–12 y) = 4 (239 ± 102.3) N (13–15 y) = 0 N (>15 y) = 1	N = 72 N (0–3 m) = 3 N (3–6 m) = 8 N (6–24 m) = 32 (578.3 ± 256.4) N (3–5 y) = 11 (391.8 ± 147.8) N (6–12 y) = 15 (453 ± 171.8) N (13–15 y) = 2 N (>15 y) = 1	NA NA 0.791 0.649 0.037 * NA NA	N = 101 N (0–3 m) = 2 N (3–6 m) = 7 N (6–24 m) = 43 (555.9 ± 226.3) N (3–5 y) = 17 (394.9 ± 149) N (6–12 y) = 24 N (13–15 y) = 2 N (>15 y) = 6	N = 15 N (0–3 m) = 2 N (3–6 m) = 3 N (6–24 m) = 4 (610.9 ± 206.8) N (3–5 y) = 5 (372.8 ± 123.9) N (6–12 y) = 1 N (13–15 y) = 0 N (>15 y) = 0	NA NA 0.642 0.767 NA NA NA	-
**HVA/5-HIAA ratio** **(mean values)** **Reference range** 1.5–3.5	N = 58 (1.12 ± 0.89)	N = 48 (1.08 ± 0.84)	0.849	N = 23 (0.84 ± 0.58)	N = 72 (1.21 ± 0.92)	0.030 *	N = 101 (1.15 ± 0.88)	N = 15 (1.10 ± 0.72)	0.816	-
**5-methyltetrahydrofolate** **(mean values)** **Reference range:** 63–111 nmol/L	N = 29 (66.89 ± 92.23)	N = 21 (53.57 ± 75.60)	0.578	N = 11 (80.57 ± 99.73)	N = 39 (76.30 ± 83.43)	0.887	N = 53 (67.57 ± 85.37)	N = 6 (53.86 ± 81.33)	0.710	-
**Biopterin** **(mean values)** **Reference range:** 2.4–9.5 µg/L	N = 57 (5.57 ± 2.07)	N = 47 (5.84 ± 3.03)	0.606	N = 23 (5.67 ± 2.84)	N = 70 (5.90 ± 2.52)	0.726	N = 103 (5.71 ± 2.40)	N = 11 (5.55 ± 4.29)	0.907	-
**Neopterin** **(mean values)** **Reference range:** 2.3–7.6 µg/L	N = 57 (1.52 ± 3.65)	N = 47 (1.62 ± 4.51)	0.908	N = 23 (1.83 ± 1.83)	N = 71 (1.92 ± 5.76)	0.909	N = 103 (1.76 ± 4.83)	N = 12 (1.07 ± 1.66)	0.312	-
**Aspartic acid** **(mean values)** **Reference range**: 0–1.57 µmol/L	N = 46 (0.86 ± 0.65)	N = 40 (0.90 ± 0.78)	0.792	N = 22 (0.76 ± 0.73)	N = 57 (0.87 ± 0.73)	0.565	N = 90 (0.98 ± 0.57)	N = 9 (0.27 ± 0.54)	<0.01 **	0.003 **
**Glycine** **(mean values)** **Reference range:** 0–10.8 µmol/L	N = 53 (4.88 ± 1.69)	N = 41 (4.53 ± 2.35)	0.427	N = 23 (4.93 ± 1.59)	N = 64 (4.51 ± 2.12)	0.337	N = 95 (4.95 ± 1.90)	N = 11 (3.63 ± 2.20)	0.034 *	0.043 *
**Leucine** **(mean values)** **Reference range**: 3.5–18.89 µmol/L	N = 53 (7.84 ± 2.68)	N = 41 (7.67 ± 3.31)	0.796	N = 23 (8.00 ± 3.08)	N = 64 (7.42 ± 2.67)	0.418	N = 95 (8.07 ± 2.73)	N = 11 (5.53 ± 3.22)	<0.01 **	0.003 **
**Ornithine** **(mean values)** **Reference range**: 1.5–10.5 µmol/L	N = 52 (6.23 ± 2.23)	N = 42 (6.25 ± 2.76)	0.962	N = 23 (6.21 ± 1.73)	N = 66 (6.07 ± 2.82)	0.781	N = 96 (6.43 ± 3.70)	N = 11 (4.85 ± 2.49)	0.045 *	0.045 *
**Serine** **(mean values)** **Reference range:** 10.4–43.39 µmol/L	N = 53 (23.92 ± 8.99)	N = 41 (23.39 ± 10.98)	0.803	N = 23 (22.60 ± 7.38)	N = 64 (24.96 ± 10.58)	0.249	N = 95 (22.78 ± 9.28)	N = 11 (29.51 ± 12.46)	0.030 *	0.043 *
**Valine** **(mean values)** Reference range: 11.9–29.39 µmol/L	N = 53 (15.53 ± 3.19)	N = 44 (16.22 ± 5.44)	0.462	N = 23 (15.14 ± 3.85)	N = 65 (15.24 ± 3.76)	0.914	N = 98 (15.98 ± 4.23)	N = 11 (13.13 ± 4.28)	0.036 *	0.043 *

**Table 3:** Relationship CSF metabolic biomarkers and features of pharmacological antiseizure treatment. The analysed metabolites that are indicated in the first column with the different age-related reference ranges, were compared within groups of patients defined by different therapeutic parameters including antiseizure medications (absent versus present), type of therapeutic schedule (mono versus polytheraphy), and drug resistance (absent versus present). The size of the groups was stratified according to the different age ranges. Legend: N = number of patients; m = months; y = years; NA = not applicable; FDR = Benjamini–Hochberg False Discovery Rate; * *p* < 0.05; ** *p* < 0.01.

Variables	ANTISEIZURE MEDICATIONS (Absent vs. Present)			ANTISEIZURE MEDICATIONS (Mono vs. Polytherapy)			DRUG-RESISTANCE (Absent vs. Present)			
Therapeutic Parameter	Absent	Present	X^2^/*p*	Monotheraphy	Polytheraphy	X^2^/*p*	Absent	Present	X^2^/*p*	FDR-Adj *p* Value
**Number of patients**	N = 40	N = 78	-	N = 46	N = 32	-	N = 54	N = 69	-	-
**Sex**	23 M + 17 F	40 M + 38 F	0.563	22 M + 24 F	18 M + 14 F	0.498	28 M + 26 F	37 M + 32 F	0.858	-
**Age (months)**	153.70 ± 61.51	171.01 ± 71.61	0.174	150.30 ± 64.26	199.88 ± 72.25	<0.01 **	161.81 ± 69.10	164.55 ± 69.05	0.828	-
**3-Methoxy-4-Hydroxyphenylglycol (mean values)** **Reference range (nmol/L)** 0–3 m = 98–168 3–6 m = 51–112 6–24 m = 28–60 3–5 y = 39–73 6–12 y = 39–73 13–15 y = 28–60 >15 y = 28–60	N = 36 N (0–3 m) = 2 N (3–6 m) = 3 N (6–24 m) = 23 (78.5 ± 50.7) N (3–5 y) = 7 (57.9 ± 21.3) N (6–12 y) = 1 N (13–15 y) = 0 N (>15 y) = 0	N = 73 N (0–3 m) = 1 N (3–6 m) = 7 N (6–24 m) = 24 (71.1 ± 32.5) N (3–5 y) = 14 (62.5 ± 18) N (6–12 y) = 20 N (13–15 y) = 2 N (>15 y) = 5	NA NA 0.556 0.628 NA NA NA	N = 41 N (0–3 m) = 2 N (3–6 m) = 2 N (6–24 m) = 21 N (3–5 y) = 7 (70.3 ± 17.5) N (6–12 y) = 6 (78.3 ± 76.7) N (13–15 y) = 1 N (>15 y) = 2	N = 30 N (0–3 m) = 0 N (3–6 m) = 5 N (6–24 m) = 0 N (3–5 y) = 7 (54.7 ± 16) N (6–12 y) = 14 (75.5 ± 39.7) N (13–15 y) = 1 N (>15 y) = 3	NA NA NA 0.108 0.916 NA NA	N = 50 N (0–3 m) = 1 N (3–6 m) = 5 N (6–24 m) = 24 (78.4 ± 49.6) N (3–5 y) = 9 (58.4 ± 21.8) N (6–12 y) = 5 (53.6 ± 11.7) N (13–15 y) = 1 N (>15 y) = 4	N = 65 N (0–3 m) = 3 N (3–6 m) = 6 N (6–24 m) = 23 (70.8 ± 33.2) N (3–5 y) = 13 (66.6 ± 20.8) N (6–12 y) = 17 (83.6 ± 54) N (13–15 y) = 1 N (>15 y) = 1	NA NA 0.543 0.401 0.044 * NA NA	-
**3-O-Methyldopa** **(mean values)** **Reference range (nmol/L)** 0–3 m = 0–300 3–6 m = 0–100 6–24 m = 0–50 3–5 y = 0–50 6–12 y = 0–50 13–15 y = 0–50 >15 y = 0–50	N = 38 N (0–3 m) = 2 N (3–6 m) = 3 N (6–24 m) = 23 (43.1 ± 30.2) N (3–5 y) = 8 (22.1 ± 5) N (6–12 y) = 2 N (13–15 y) = 0 N (>15 y) = 0	N = 78 N (0–3 m) = 1 N (3–6 m) = 7 N (6–24 m) = 26 (34.3 ± 19.5) N (3–5 y) = 14 (29.3 ± 22.9) N (6–12 y) = 22 N (13–15 y) = 2 N (>15 y) = 6	NA NA 0.238 0.395 NA NA NA	N = 43 N (0–3 m) = 2 N (3–6 m) = 2 N (6–24 m) = 23 N (3–5 y) = 7 (36.9 ± 24.6) N (6–12 y) = 7 (108.9 ± 210.8) N (13–15 y) = 1 N (>15 y) = 2	N = 32 N (0–3 m) = 0 N (3–6 m) = 5 N (6–24 m) = 0 N (3–5 y) = 7 (21.6 ± 19.9) N (6–12 y) = 15 (41 ± 66.1) N (13–15 y) = 1 N (>15 y) = 4	NA NA NA 0.226 0.083 NA NA	N = 51 N (0–3 m) = 1 N (3–6 m) = 5 N (6–24 m) = 24 (43.8 ± 29.7) N (3–5 y) = 9 (29.1 ± 22.6) N (6–12 y) = 6 (23.9 ± 7.8) N (13–15 y) = 1 N (>15 y) = 5	N = 68 N (0–3 m) = 3 N (3–6 m) = 6 N (6–24 m) = 25 (33.3 ± 19.2) N (3–5 y) = 13 (24.6 ± 14.6) N (6–12 y) = 19 (67.7 ± 138.7) N (13–15 y) = 1 N (>15 y) = 1	NA NA 0.151 0.569 0.454 NA NA	-
**5-Hydroxytryptophan** **(mean values)** **Reference range (nmol/L)** 0–3 m = 0–10 3–6 m = 0–10 6–24 m = 0–10 3–5 y = 0–10 6–12 y = 0–10 13–15 y = 0–10 >15 y = 0–10	N = 32 N (0–3 m) = 2 N (3–6 m) = 3 N (6–24 m) = 21 (7.2 ± 3.5) N (3–5 y) = 7 (6.1 ± 2.7) N (6–12 y) = 2 N (13–15 y) = 0 N (>15 y) = 0	N = 72 N (0–3 m) = 1 N (3–6 m) = 7 N (6–24 m) = 26 (6.7 ± 2.6) N (3–5 y) = 13 (6.5 ± 3.4) N (6–12 y) = 20 N (13–15 y) = 2 N (>15 y) = 6	NA NA 0.563 0.795 NA NA NA	N = 41 N (0–3 m) = 2 N (3–6 m) = 2 N (6–24 m) = 21 N (3–5 y) = 5 (6.2 ± 3.3) N (6–12 y) = 7 (17.9 ± 24) N (13–15 y) = 1 N (>15 y) = 2	N = 30 N (0–3 m) = 0 N (3–6 m) = 5 N (6–24 m) = 0 N (3–5 y) = 7 (6.8 ± 3.7) N (6–12 y) = 13 (17.8 ± 30.9) N (13–15 y) = 1 N (>15 y) = 4	NA NA NA 0.745 0.991 NA NA	N = 50 N (0–3 m) = 1 N (3–6 m) = 5 N (6–24 m) = 22 (7.2 ± 3.4) N (3–5 y) = 10 (6.6 ± 3.1) N (6–12 y) = 6 (5.7 ± 2) N (13–15 y) = 1 N (>15 y) = 5	N = 65 N (0–3 m) = 3 N (3–6 m) = 6 N (6–24 m) = 25 (6.6 ± 2.6) N (3–5 y) = 12 (6 ± 3.2) N (6–12 y) = 17 (19.7 ± 30) N (13–15 y) = 1 N (>15 y) = 1	NA NA 0.506 0.631 0.274 NA NA	-
**5-hydroxyindoleacetic acid (5-HIAA)** **(mean values)** **Reference range (nmol/L)** 0–3 m = 189–1380 3–6 m = 152–462 6–24 m = 97–367 3–5 y = 89–341 6–12 y = 68–220 13–15 y = 68–115 >15 y = 45–135	N = 35 N (0–3 m) = 2 N (3–6 m) = 3 N (6–24 m) = 23 (231.3 ± 110.2) N (3–5 y) = 8 (141.3 ± 42.6) N (6–12 y) = 2 N (13–15 y) = 0 N (>15 y) = 0	N = 75 N (0–3 m) = 1 N (3–6 m) = 7 N (6–24 m) = 26 (223.1 ± 114.6) N (3–5 y) = 14 (140.4 ± 52.3) N (6–12 y) = 22 N (13–15 y) = 2 N (>15 y) = 6	NA NA 0.799 0.968 NA NA NA	N = 44 N (0–3 m) = 2 N (3–6 m) = 2 N (6–24 m) = 23 N (3–5 y) = 7 (168.5 ± 46.2) N (6–12 y) = 7 (142.1 ± 72) N (13–15 y) = 1 N (>15 y) = 2	N = 32 N (0–3 m) = 0 N (3–6 m) = 5 N (6–24 m) = 0 N (3–5 y) = 7 (112.3 ± 44) N (6–12 y) = 15 (159.9 ± 69.7) N (13–15 y) = 1 N (>15 y) = 4	NA NA NA 0.038 * 0.596 NA NA	N = 52 N (0–3 m) = 1 N (3–6 m) = 5 N (6–24 m) = 24 (234.3 ± 108.8) N (3–5 y) = 10 (152.9 ± 40.1) N (6–12 y) = 6 (120.4 ± 46.3) N (13–15 y) = 1 N (>15 y) = 5	N = 68 N (0–3 m) = 3 N (3–6 m) = 6 N (6–24 m) = 25 (219.8 ± 115.7) N (3–5 y) = 13 (132.6 ± 50.9) N (6–12 y) = 19 (161.8 ± 70.5) N (13–15 y) = 1 N (>15 y) = 1	NA NA 0.652 0.316 0.274 NA NA	-
**Homovanillic** **acid (HVA)** **(mean values)** **Reference range (nmol/L)** 0–3 m = 324–1379 3–6 m = 302–845 6–24 m = 236–867 3–5 y = 231–840 6–12 y = 137–582 13–15 y = 148–434 >15 y = 98–450	N = 35 N (0–3 m) = 2 N (3–6 m) = 3 N (6–24 m) = 23 (533.9 ± 154.6) N (3–5 y) = 8 (405.9 ± 123.5) N (6–12 y) = 2 N (13–15 y) = 0 N (> 15 y) = 0	N = 75 N (0–3 m) = 1 N (3–6 m) = 7 N (6–24 m) = 26 (605.3 ± 274.4) N (3–5 y) = 14 (381.8 ± 154.9) N (6–12 y) = 22 N (13–15 y) = 2 N (>15 y) = 6	NA NA 0.262 0.711 NA NA NA	N = 45 N (0–3 m) = 2 N (3–6 m) = 2 N (6–24 m) = 23 N (3–5 y) = 7 (441.1 ± 138.8) N (6–12 y) = 7 (351.6 ± 151.8) N (13–15 y) = 1 N (>15 y) = 2	N = 32 N (0–3 m) = 0 N (3–6 m) = 5 N (6–24 m) = 0 N (3–5 y) = 7 (322.4 ± 156.5) N (6–12 y) = 15 (430.1 ± 177.4) N (13–15 y) = 1 N (>15 y) = 4	NA NA NA 0.159 0.325 NA NA	N = 52 N (0–3 m) = 1 N (3–6 m) = 5 N (6–24 m) = 24 (540.8 ± 154.9) N (3–5 y) = 10 (402.8 ± 154.2) N (6–12 y) = 6 (325.9 ± 105.1) N (13–15 y) = 1 N (>15 y) = 5	N = 68 N (0–3 m) = 3 N (3–6 m) = 6 N (6–24 m) = 25 (601.6 ± 279.4) N (3–5 y) = 13 (388 ± 133.7) N (6–12 y) = 19 (427.4 ± 174.8) N (13–15 y) = 1 N (>15 y) = 1	NA NA 0.350 0.808 0.193 NA NA	-
**HVA/5-HIAA ratio** **(mean values)** **Reference range** 1,5–3,5	N = 35 (1.02 ± 0.68)	N = 75 (1.22 ± 0.94)	0.201	N = 44 (1.27 ± 0.88)	N = 32 (1.16 ± 1.02)	0.638	N = 52 (0.99 ± 0.73)	N = 68 (1.26 ± 0.94)	0.085	-
**Methyltetrahydrofolate** **(mean values)** **Reference range:** 63–111 nmol/L	N = 22 (98.07 ± 92.47)	N = 37 (47.22 ± 74.13)	0.024 *	N = 24 (59.03 ± 76.73)	N = 13 (25.40 ± 66.40)	0.175	N = 30 (72.61 ± 90.64)	N = 29 (59.53 ± 78.44)	0.556	-
**Biopterin** **(mean values)** Reference range: 2.3–7.6 µg/L	N = 38 (6.04 ± 2.50)	N = 78 (5.36 ± 2.34)	0.166	N = 46 (5.52 ± 2.24)	N = 32 (5.14 ± 2.49)	0.492	N = 52 (5.70 ± 2.68)	N = 67 (5.59 ± 2.54)	0.820	-
**Neopterin** **(mean values)** **Reference range:** 2.4–9.5 µg/L	N = 38 (1.17 ± 4.69)	N = 79 (1.81 ± 4.58)	0.650	N = 46 (1.78 ± 4.56)	N = 33 (1.84 ± 4.68)	0.956	N = 52 (1.84 ± 5.70)	N = 68 (1.50 ± 3.44)	0.700	-
**2-Aminobutyric acid** **Reference range:** 0–5.09 µmol/L	N = 36 (1.17 ± 1.17)	N = 71 (0.49 ± 2.05)	0.033 *	N = 42 (0.64 ± 1.81)	N = 29 (0.27 ± 2.37)	0.483	N = 49 (0.99 ± 1.46)	N = 59 (0.50 ± 2.05)	0.497	0.497
**Glutamine** **Reference range:** 231–765 µmol/L	N = 36 (287.19 ± 67.57)	N = 71 (208.53 ± 133.66)	<0.01 **	N = 42 (234.96 ± 103.10)	N = 29 (170.26 ± 162.90)	0.044 *	N = 49 (260.33 ± 93.38)	N = 59 (213.71 ± 136.57)	0.038 *	0.076

**Table 4:** Relationships between CSF metabolic biomarkers and occurrence of movement and neurodevelopmental disorders. The analysed metabolites that are indicated in the first column with the different age-related reference ranges, were compared within groups of patients defined by the presence of the main comorbidities represented by movement and neurodevelopmental disorders (absent versus present) The size of the groups was stratified according to the different age ranges. Legend: N = number of patients; m = months; y = years; NA = not applicable; FDR = Benjamini–Hochberg False Discovery Rate; * *p* < 0.05; ** *p* < 0.01.

Variables	MOVEMENT DISORDERS (Absent vs. Present)			NEURODEVELOPMENTAL DISORDERS (Absent vs. Present)			
Presence of Comorbidity	Absent	Present	X^2^/*p*	Absent	Present	X^2^/*p*	FDR-Adj *p* Value
**Number of patients**	N = 63	N = 60	-	N = 16	N = 101	-	-
**Sex**	34 M + 29 F	31 M + 29 F	0.857	5 M + 11 F	57 M + 44 F	0.104	-
**Age (months)**	164.52 ± 61.40	160.77 ± 75.94	0.765	162.81 ± 58.60	164.43 ± 71.41	0.922	-
**3-Methoxy-4-Hydroxyphenylglycol (mean values)** **Reference range (nmol/L)** 0–3 m = 98–168 3–6 m = 51–112 6–24 m = 28–60 3–5 y = 39–73 6–12 y = 39–73 13–15 y = 28–60 >15 y = 28–60	N = 56 N (0–3 m) = 1 N (3–6 m) = 8 N (6–24 m) = 28 (71.6 ± 42.7) N (3–5 y) = 9 (74 ± 15.1) N (6–12 y) = 6 (84.6 ± 67.7) N (13–15 y) = 2 N (>15 y) = 2	N = 56 N (0–3 m) = 3 N (3–6 m) = 3 N (6–24 m) = 19 (79.3 ± 41.9) N (3–5 y) = 13 (57 ± 22) N (6–12 y) = 15 (73.9 ± 39.7) N (13–15 y) = 0 N (>15 y) = 3	NA NA 0.537 0.050 * 0.645 NA NA	N = 13 N (0–3 m) = 0 N (3–6 m) = 0 N (6–24 m) = 7 (99.8 ± 77.7) N (3–5 y) = 4 (68.6 ± 17.1) N (6–12 y) = 1 N (13–15 y) = 1 N (>15 y) = 0	N = 96 N (0–3 m) = 4 N (3–6 m) = 10 N (6–24 m) = 36 (68.4 ± 29.4) N (3–5 y) = 18 (62.2 ± 22.1) N (6–12 y) = 22 N (13–15 y) = 1 N (>15 y) = 5	NA NA 0.331 0.596 NA NA NA	-
**3-O-Methyldopa** **(mean values)** **Reference range (nmol/L)** 0–3 m = 0–300 3–6 m = 0–100 6–24 m = 0–50 3–5 y = 0–50 6–12 y = 0–50 13–15 y = 0–50 >15 y = 0–50	N = 60 N (0–3 m) = 1 N (3–6 m) = 8 N (6–24 m) = 28 (40.5 ± 28.8) N (3–5 y) = 10 (22.6 ± 7.2) N (6–12 y) = 8 (30.6 ± 24.4) N (13–15 y) = 2 N (>15 y) = 3	N = 57 N (0–3 m) = 3 N (3–6 m) = 3 N (6–24 m) = 19 (35.8 ± 19.7) N (3–5 y) = 13 (29.7 ± 23.3) N (6–12 y) = 16 (73.2 ± 150.4) N (13–15 y) = 0 N (>15 y) = 3	NA NA 0.524 0.363 0.440 NA NA	N = 14 N (0–3 m) = 0 N (3–6 m) = 0 N (6–24 m) = 7 (39.9 ± 21.7) N (3–5 y) = 4 (47.2 ± 29.8) N (6–12 y) = 1 N (13–15 y) = 1 N (>15 y) = 1	N = 101 N (0–3 m) = 4 N (3–6 m) = 10 N (6–24 m) = 37 (36 ± 19.4) N (3–5 y) = 19 (22.2 ± 11.9) N (6–12 y) = 25 N (13–15 y) = 1 N (>15 y) = 5	NA NA 0.631 0.009 ** NA NA NA	-
**5-Hydroxytryptophan** **(mean values)** **Reference range (nmol/L)** 0–3 m = 0–10 3–6 m = 0–10 6–24 m = 0–10 3–5 y = 0–10 6–12 y = 0–10 13–15 y = 0–10 >15 y = 0–10	N = 57 N (0–3 m) = 1 N (3–6 m) = 8 N (6–24 m) = 26 (6.6 ± 2.9) N (3–5 y) = 10 (7 ± 3.2) N (6–12 y) = 7 (8.6 ± 4.8) N (13–15 y) = 2 N (>15 y) = 3	N = 54 N (0–3 m) = 3 N (3–6 m) = 3 N (6–24 m) = 19 (7.3 ± 3.2) N (3–5 y) = 11 (5.6 ± 3.1) N (6–12 y) = 15 (20.2 ± 32.2) N (13–15 y) = 0 N (>15 y) = 3	NA NA 0.369 0.329 0.359 NA NA	N = 12 N (0–3 m) = 0 N (3–6 m) = 0 N (6–24 m) = 6 (7.1 ± 3) N (3–5 y) = 3 (5.9 ± 1.5) N (6–12 y) = 1 N (13–15 y) = 1 N (>15 y) = 1	N = 98 N (0–3 m) = 4 N (3–6 m) = 10 N (6–24 m) = 37 (6.6 ± 2.8) N (3–5 y) = 18 (6.3 ± 3.3) N (6–12 y) = 23 N (13–15 y) = 1 N (>15 y) = 5	NA NA 0.640 0.853 NA NA NA	-
**-5-hydroxyindoleacetic acid (5-HIAA)** **(mean values)** **Reference range (nmol/L)** 0–3 m = 189–1380 3–6 m = 152–462 6–24 m = 97–367 3–5 y = 89–341 6–12 y = 68–220 13–15 y = 68–115 >15 y = 45–135	N = 60 N (0–3 m) = 1 N (3–6 m) = 8 N (6–24 m) = 28 (218.2 ± 99.1) N (3–5 y) = 10 (159.8 ± 57.2) N (6–12 y) = 8 (162 ± 49.7) N (13–15 y) = 2 N (>15 y) = 3	N = 57 N (0–3 m) = 3 N (3–6 m) = 3 N (6–24 m) = 19 (238.6 ± 127.7) N (3–5 y) = 13 (127.8 ± 57.2) N (6–12 y) = 16 (146.8 ± 77.2) N (13–15 y) = 0 N (>15 y) = 3	NA NA 0.532 0.102 0.619 NA NA	N = 14 N (0–3 m) = 0 N (3–6 m) = 0 N (6–24 m) = 7 (192.2 ± 59.5) N (3–5 y) = 4 (164.1 ± 22.8) N (6–12 y) = 1 N (13–15 y) = 1 N (>15 y) = 1	N = 101 N (0–3 m) = 4 N (3–6 m) = 10 N (6–24 m) = 37 (220.2 ± 100.3) N (3–5 y) = 19 (136.7 ± 49.7) N (6–12 y) = 25 N (13–15 y) = 1 N (>15 y) = 5	NA NA 0.481 0.298 NA NA NA	-
**Homovanillic** **acid (HVA)** **(mean values)** **Reference range (nmol/L)** 0–3 m = 324–1379 3–6 m = 302–845 6–24 m = 236–867 3–5 y = 231–840 6–12 y = 137–582 13–15 y = 148–434 >15 y = 98–450	N = 60 N (0–3 m) = 1 N (3–6 m) = 8 N (6–24 m) = 28 (578.2 ± 174.1) N (3–5 y) = 10 (423.2 ± 148) N (6–12 y) = 8 (449.2 ± 118.6) N (13–15 y) = 2 N (>15 y) = 3	N = 57 N (0–3 m) = 3 N (3–6 m) = 3 N (6–24 m) = 19 (563.3 ± 287.2) N (3–5 y) = 13 (372.4 ± 134.7) N (6–12 y) = 16 (384.7 ± 187.2) N (13–15 y) = 0 N (>15 y) = 3	NA NA 0.822 0.399 0.387 NA NA	N = 14 N (0–3 m) = 0 N (3–6 m) = 0 N (6–24 m)= 7 (489.36 ± 182.1) N (3–5 y) = 4 (459.4 ± 118.4) N (6–12 y) = 1 N (13–15 y) = 1 N (>15 y) = 1	N = 101 N (0–3 m) = 4 N (3–6 m) = 10 N (6–24 m) = 37 (582.7 ± 233.3) N (3–5 y) = 19 (380.8 ± 142.8) N (6–12 y) = 25 N (13–15 y) = 1 N (>15 y) = 5	NA NA 0.324 0.318 NA NA NA	-
**HVA/5-HIAA ratio** **(mean values)** **Reference range** 1,5–3,5	N = 60 (1.27 ± 0.93)	N = 57 (1.02 ± 0.79)	0.112	N = 14 (1.04 ± 0.66)	N = 101 (1.18 ± 0.89)	0.463	-
**Methyltetrahydrofolate** **(mean values)** **Reference range:** 63–111 nmol/L	N = 34 (90.29 ± 87.43)	N = 25 (33.40 ± 68.98)	<0.01 **	N = 9 (60.20 ± 71.07)	N = 49 (63.73 ± 84.39)	0.897	-
**Biopterin** **(mean values)** **Reference range:** 2.3–7.6 µg/L	N = 60 (6.26 ± 2.76)	N = 58 (5.05 ± 2.25)	0.010 *	N = 15 (6.10 ± 2.27)	N = 98 (5.55 ± 2.68)	0.401	-
**Neopterin** **(mean values)** **Reference range:** 2.4–9.5 µg/L	N = 60 (1.83 ± 4.99)	N = 59 (1.48 ± 4.11)	0.677	N = 15 (4.94 ± 9.61)	N = 99 (1.25 ± 3.14)	0.161	-
**Aspartic acid** **(mean values)** **Reference range:** 0–1.57 µmol/L	N = 52 (0.79 ± 0.62)	N = 48 (0.93 ± 0.80)	0.329	N = 11 (0.45 ± 0.54)	N = 85 (0.91 ± 0.70)	0.022 *	0.022 *
**Proline** **Reference range:** 0–15 µmol/L	N = 54 (12.32 ± 2.36)	N = 45 (12.84 ± 1.82)	0.218	N = 14 (11.31 ± 3.32)	N = 80 (12.78 ± 1.77)	0.016 *	0.022 *

## Data Availability

Data are available upon specific request addressed to the authors due to privacy restrictions.

## Supplementary Materials

The following supporting information can be downloaded at https://www.mdpi.com/article/10.3390/children12111514/s1. Table S1: Demographic data, molecular genetic features and epilepsy phenotype of the reported cohort.

## Abbreviations

CSF	cerebrospinal Fluid
HVA	Homovanillic Acid
HVA/(5HIAA) Ratio	Homovanillic Acid/5-Hydroxyindolacetic Acid
3-OMD	3-O-methyldopa
5-MTHF	5-Methyltetrahydrofolate
3-MHPG	3-methoxy-4-hydroxyphenylglycol
